# Recurrent syncope, orthostatic hypotension and volatile hypertension: think outside the box

**DOI:** 10.3402/jchimp.v3i2.20741

**Published:** 2013-07-05

**Authors:** Thein Aung, Wuqiang Fan, Mahesh Krishnamurthy

**Affiliations:** 1 Department of Internal Medicine, Easton Hospital (Academic Affiliate Drexel University), Easton, PA, USA; 2 Internal Medicine Residency Program, Easton Hospital (Academic Affiliate Drexel University), Easton, PA, USA; 3 Geriatric Medical Education, Division of Geriatrics, Drexel University, Philadelphia, PA, USA

**Keywords:** baroreflex failure, baroreceptors, carotid sinus, orthostatic hypotension

## Abstract

The baroreceptors in the neck and aortic arch are important regulators of sudden blood pressure changes. They are innervated by CN IX and X and synapse in the brainstem. Baroreceptor failure is an under-recognized cause of recurrent syncope, orthostatic hypotension, and volatile hypertension, which is refractory to and may in fact worsen with conventional treatments. Baroreflex failure can be the result of neck and chest radiation, head and neck surgery, and cerebrovascular accidents involving the brainstem nuclei. The management of baroreflex failure is a challenge since patient education, lifestyle changes, and family support are extremely important in managing blood pressure. Leg exercises and Thrombo-Embolic Deterrent Stockings (TED) stockings are important in treating orthostatic hypotension. Clonidine is the antihypertensive of choice for supine hypertension. Low-dose benzodiazepines are helpful in suppressing sympathetic surges. We have encountered two patients with baroreflex failure after chemotherapy and radiation to the neck or upper chest. Temporal relationship between symptoms onset and the history of head, neck, and upper chest radiation or trauma is important in reaching a diagnosis.

Baroreceptors monitor sudden changes in blood pressure and buffer these changes to prevent excessive fluctuations (rise or fall) in blood pressure. Baroreflex failure can lead to significant volatility in blood pressure values leading to either orthostatic hypotension or supine hypertension. It can present as recurrent syncope, which is refractory to conventional treatments. Without understanding the pathophysiology behind the baroreflex failure, managing these patients can be challenging. We have encountered two patients with baroreflex failure after chemotherapy and radiation to the neck or upper chest (1).

## Case 1

A 74-year-old male presented with multiple episodes of lightheadedness and the feeling of ‘passing out’ while he was getting out of his bed and walking. He had a 40-pack-year smoking history, and his past medical history was significant for nasopharyngeal cancer for which he received radiation and chemotherapy 6 years earlier. He had no history of diabetes or hypertension. Examination revealed significant orthostatic changes in blood pressure, 168/100 mmHg upon lying down and 102/44 mmHg upon standing up. The rest of his physical exam was negative. Extensive laboratory, cardiac, and neurological testing came back within normal limits. His symptoms did not improve with intravenous fluids. Normal saline infusion worsened his supine hypertension.

A diagnosis of baroreceptor failure was made on the basis of the history of neck radiation, tilt table test, depressor response (hypotension and widened pulse pressure response) to a small dose of clonidine. Tilt table testing showed progressive orthostatic hypotension without a significant change in heart rate. A variety of non-pharmacologic measures such as increasing dietary salt and caffeine, TED stockings and leg muscle exercising after prolonged standing were recommended to decrease fluctuations in blood pressure. He was treated with low doses of alprazolam, clonidine, and fludrocortisone. On follow-up, the patient felt better but still had occasional postural lightheadedness.

## Case 2

A 58-year-old Caucasian male was found to have a large right upper lung mass, which was subsequently confirmed to be small cell carcinoma of the lung by endobronchial biopsy. The cancer was complicated with superior vena cava (SVC) syndrome. The patient then received two cycles of Cisplatin and Etoposide-based chemotherapy concurrently with a radiation therapy to the lower neck, right lung, and mediastinum. The radiotherapy was stopped at a total dose of 4,640 cGy because of the development of a significant esophagitis. He also became hypertensive with severe tachycardia. His hypertension resolved with medication; however, he later developed persistent orthostatic hypotension that was refractory to intravenous fluids. His blood pressure was 130/71 at supine level, 113/48 upon sitting up, and 73/46 upon standing up. The rest of his physical exam as well as extensive laboratory, cardiac testing were normal. Normal cortisol levels ruled out adrenal insufficiency. A diagnosis of baroreceptor failure was made based on the history of extensive radiotherapy. The patient was started on a liberal sodium diet, midodrine, fludrocortisone, and TED stockings. He was also advised to do physical activities such as leg exercises to decrease fluctuations in blood pressure. His orthostatic hypotension has since improved on follow-up.

## Discussion

Baroreceptors are mechanoreceptor sensory neurons that are excited by stretching of the corresponding blood vessel. They act through an instantaneous negative feedback response system called baroreflex to buffer huge changes in blood pressure (2).

Baroreceptors can be subclassified into high-pressure arterial baroreceptors and low-pressure volume receptors or cardiopulmonary receptors. Among the arterial baroreceptors, the most important are carotid sinus baroreceptors and aortic arch baroreceptors. The carotid baroreceptors are located in carotid sinuses, close to the bifurcation of the internal carotid artery from the common carotid artery. They are innervated by the sinus nerve of Hering, a branch of the glossopharyngeal nerve (CN IX). Aortic arch baroreceptors can be found in the tunica adventitia of the aorta in the aortic arch and innervated by the vagus nerve (CN X). They synapse in the nucleus tractus solitarus, which in turn modulates activity of autonomic nervous system. Both sympathetic and parasympathetic pathways serve as the efferent pathway. The effectors of baroreceptor reflex include: sino-atrial node, atrioventricular node, cardiac muscle cells, and arterial and venous smooth muscles.

Standing up from a lying position can cause a significant reduction in blood volume as 300–800 ml blood pools into the lower extremities. This drastic drop of venous return leads to decreased venous return, decreased cardiac output, hypotension, and inadequate perfusion of vital organs. In response to hypotension, the sympathetic nervous system is activated. Peripheral vasoconstriction by catecholamine release from nerve endings lead to increased venous return, and ultimately, increased preload. On the cardiac muscles and nodal cells, it has inotropic, chronotropic, and dromotropic effects. It augments cardiac conduction, contractility, and heart rate, leading to increased stroke volume and cardiac output. All of these actions ultimately restore blood pressure and perfusion to the vital organs (2).

Baroreflex failure can occur after neck radiation, surgery and cerebrovascular accidents involving the brainstem nuclei, and results in improper regulation of autonomic neurons that leads to impaired blood pressure regulation. Neck irradiation can be complicated with a late-onset of baroreflex failure (3). Despite being well documented in the medical literature, baroreflex failure remains an under-diagnosed cause of labile hypertension with orthostatic changes (4). While damage of carotid sinus is an established cause of baroreflex failure, our second case suggests that radiation-related damage of the baroreceptors in the aortic arch may have similar consequences.

Baroreflex can be measured by methods involving the application of vasoactive drugs (i.e., phenylephrine), Valsalva maneuver, and mechanical manipulation by the neck chamber technique that produces a negative/positive local pressure for the baroreceptors in the neck region (5). These techniques are mostly experimental and rarely used in clinical practice since excessive stimulation of baroreceptors can lead to a sudden drop in systolic blood pressure and can lead to disastrous complications.

Patients with baroreflex failure typically present with postural lightheadedness, orthostatic hypotension, syncope and also labile hypertension, which can also be found in autonomic nervous system failure. In fact, baroreflex failure is a part of the spectrum of autonomic nervous system failure and typically presents with systolic and diastolic hypertensive episodes, tachycardia, and hypotension alternating with relative bradycardia at rest (6).

Differential diagnosis for orthostatic hypotension in these cases include paraneoplastic autonomic neuropathy (PNAN), which is most often seen in patients with small cell lung cancer. The prominent clinical findings of PNAN other than orthostatic hypotension include bowel hypomotility, intestinal obstruction, bladder dysfunction, pupillomotor and sudomotor dysfunction, and xerophthalmia. Both our patients did not have any other signs of autonomic dysfunction other than orthostatic hypotension.

Although PNAN is often associated with anti-Hu antibodies and anti-CV2/CRMP-5 antibodies (7), they were not tested in the above-mentioned patients. The clinical presentations of orthostatic hypotension without any other signs of autonomic failure make the diagnosis of PNAN unlikely. However, we do feel that it would have been better to have checked these antibodies in our patients and in any future patients presenting with similar clinical features.

We made the diagnosis of baroreflex failure based on the clinical picture of orthostatic hypotension, past medical history of head, neck and/or upper chest radiation, normal electrolytes, and the systematic exclusion of other possible diagnoses. Tilt table test showed progressive orthostatic hypotension and no significant change of heart rate. These findings excluded vasovagal and reflex syncope as the possible cause of syncope as bradycardia and hypotension are the expected findings.

The time of onset can vary from patient to patient based on the volume status, baseline activities, family support, and co-morbid conditions such as hypertension, diabetes, arthritis, and dementia. The first patient had recurrent syncope attacks but tolerated the symptoms for several years before coming to the hospital. With the second patient, the symptoms were of sudden onset and severe enough to seek more prompt medical attention.

Baroreflex failure is a therapeutic challenge since both the hypertension and hypotension are often difficult to control with conventional therapy. In addition, many medications that do not elicit blood pressure changes in healthy subjects may have a dramatic effect in baroreflex failure patients (8). Drugs that can increase norepinephrine secretion or reuptake blockers are contraindicated in baroreflex failure since they can induce hypertensive episodes. Those include tricyclic antidepressants; amphetamines; monoamine oxidase A inhibitors; and food and beverages containing tyramine, cocaine, and prednisone (9).

Management typically includes non-pharmacological and pharmacological components. The first and foremost step of non-pharmacological measures should be education of the patient and his/her family about the importance of measuring his/her blood pressure frequently. Second, timing of food and water should be carefully adjusted since carbohydrate ingestion can lower blood pressure by 30–40 mmHg. It can be useful in managing supine hypertension at night. Drinking water also has a significant, dose dependent, pressor effect in these patients. Sixteen ounces of water raised the systemic blood pressure by 20–40 mmHg in a clinical trial presented by Jordan et al. (10). Oral fluids have shown a significant pressor effect in clinical trials, it was thought easier to give intravenous fluids in the in-patient setting when the patients were admitted. In our patients, both oral and intravenous fluids improved the systemic blood pressure but some orthostatic hypotension still persisted. Both patients were advised to continue drinking lots of liquids when they were discharged to the out-patient setting. Caffeine ingestion with meals is helpful in some patients by increasing the blood pressure. Finally, physical maneuvers and exercises such as entwining the legs, constricting leg muscles, raising one foot to the seat of a chair, or squatting can counteract loss of sympathetic tone. Swimming can be an excellent treatment since water can effectively compress lower extremities and increase venous return. Elevating the head of the bed up to eight inches at nighttime can reduce episodes of nocturia and prevent volume depletion in the morning (11).

Pharmacologically, clonidine, a centrally acting alpha-2 agonist, is the most effective agent and hence the antihypertensive of choice. It works centrally and peripherally to attenuate sympathetic activation and limit the hypertensive episodes (9). In the study done by Robertson et al., clonidine had reduced the frequency and severity of tachycardia and hypertensive episodes by 81% (1). Low-dose benzodiazepines are also helpful in suppressing sympathetic surges.

Fludrocortisone can cause salt and water retention and alleviate orthostatic hypotension. In order to see the mineralocorticoids effect, patients should be encouraged to consume a salt-liberal diet or take a gram of salt tablet at breakfast and lunch.

## Conclusions

Baroreflex failure following radiotherapy is an under-recognized cause of labile blood pressures with orthostatic changes and recurrent syncope. Diagnosis can be challenging since the majority of signs and symptoms overlap with those of autonomic failure. Timing of symptoms onset and a history of head, neck or chest radiation are important hints. A clear temporal relationship can make the diagnostic process relatively straight forward whereas a huge time gap between radiation and symptoms onset can obscure the diagnosis. Radiation is a common cause of baroreceptor damage. While neck irradiation is a well-recognized cause, intensive upper chest irradiation may have similar consequences due to damage to the baroreceptors in the aortic arch. Both the hypertension and hypotension are often difficult to control with conventional therapy. Clonidine is the antihypertensive of choice for supine hypertension. Low-dose benzodiazepines are helpful in suppressing sympathetic surges. Physical maneuvers like periodic squatting help maintain blood pressure when upright. Fludrocortisone, midodrine and TED stocking are the treatments of choice for orthostatic hypotension.
